# Clinical Features and Correlates of Excessive Daytime Sleepiness in Parkinson's Disease

**DOI:** 10.3389/fneur.2019.00121

**Published:** 2019-02-19

**Authors:** Ya-qin Xiang, Qian Xu, Qi-ying Sun, Zhi-qin Wang, Yun Tian, Liang-juan Fang, Yang Yang, Jie-qiong Tan, Xin-xiang Yan, Bei-sha Tang, Ji-feng Guo

**Affiliations:** ^1^Department of Neurology, Xiangya Hospital, Central South University, Changsha, China; ^2^Laboratory of Medical Genetics, Central South University, Changsha, China; ^3^National Clinical Research Center for Geriatric Disorders, Changsha, China; ^4^Key Laboratory of Hunan Province in Neurodegenerative Disorders, Central South University, Changsha, China; ^5^Collaborative Innovation Center for Brain Science, Shanghai, China; ^6^Parkinson's Disease Center of Beijing Institute for Brain Disorders, Beijing, China; ^7^Collaborative Innovation Center for Genetics and Development, Shanghai, China

**Keywords:** excessive daytime sleepiness, Parkinson's disease, sleep disorders, clinical features, risk factors

## Abstract

**Objective:** To explore the clinical features and correlates of excessive daytime sleepiness (EDS) in a Chinese population of Parkinson's disease (PD) patients.

**Methods:** Patients with clinically established or clinically probable PD were recruited. Clinical and demographic data were collected, and participants were evaluated using standardized assessment protocols. Patients were divided into PD with EDS and PD without EDS groups based on the Epworth sleepiness scale (ESS) scores, with a cutoff score of 10. Clinical manifestations were compared between patients with and without EDS, and correlates of EDS were also studied. In addition, the relationship between EDS and poor nighttime sleep quality was analyzed.

**Results:** A total of 1,221 PD patients were recruited in our study. The mean ESS (min, max) score was 7.6 ± 6.1 (0, 24), and 34.1% of the patients had ESS scores ≥10. No difference was seen in lifestyle (except for alcohol consumption), environmental factors, BMI, levodopa equivalent dose (LED), initial presentation of motor symptoms, motor subtype, and wearing off between patients with and without EDS. The PD with EDS group had a higher proportion of male patients and a higher average patient age. Moreover, the PD with EDS group showed older age at PD onset, lower educational level, and longer disease duration. Patients with EDS had higher scores on the Hoehn-Yahr scale and the Unified Parkinson's Disease Rating Scale (UPDRS) parts I, II, and III score, more severe non-motor symptoms, and poorer quality of sleep and life. Logistic regression analyses demonstrated that EDS was associated with male sex, age, cognitive impairment, PD-related sleep problems, rapid eye movement sleep behavior disorder (RBD), and worse quality of life (QoL).

**Conclusion:** EDS is a general clinical manifestation in PD, and there were significant differences in clinical features between patients with and without EDS. Moreover, our study proved that many factors were associated with EDS, including male sex, age, cognitive impairment, PD-related sleep problems, RBD, and worse QoL. Understanding the clinical characteristics of EDS in PD patients may help identify EDS early, improve QoL, and reduce the occurrence of accidents.

## Introduction

Parkinson's disease (PD) is a common neurodegenerative disease. While environmental exposure and genetic background are thought to be major risk factors for PD (1, 2), the etiology of the disease is not fully understood. PD is characterized by classical motor features including tremor, rigidity, bradykinesia, and postural instability. However, the clinical manifestations of PD are identified as heterogeneous, and the disease presents with a wide range of non-motor symptoms (NMS) (3, 4).

Excessive daytime sleepiness (EDS) is one of the common NMS in PD. The most significant clinical manifestation of EDS is an inability to stay awake and alert during a major part of the day. The reported incidence of EDS varies widely due to differences in sample sizes, assessment methods, and diagnostic criteria for sleep disorders. A cross-sectional study of 436 PD patients indicated that the incidence of EDS was 37.8% (5), while in another study, the incidence of EDS was 26.3% (6). Moreover, the prevalence of EDS in PD patients increases with longer disease duration. Zhu et al revealed that 43% of PD patients had EDS at baseline, while 46% of patients without EDS at baseline developed EDS after 5 years (7). Tholfsen et al indicated that the incidence of PD with EDS increased from 11.8% at baseline to 23.4% after 5 years' follow-up (8). The etiology of EDS in PD is complicated and multifactorial. Except for disease-related pathological changes, many variables have been revealed to be associated with EDS in PD, including male gender, poorer nighttime sleep, cognitive impairment, autonomic dysfunction, hallucinations, depression, anxiety, advanced disease, non-tremor dominant motor phenotype, dosage of dopamine agonists, and use of anti-hypertensives (7–11). Because of the diversity in sample sizes and research methods, the results of previous studies on EDS-associated factors were inconsistent.

EDS seriously influences patients' quality of life (QoL) and increases the risk of accidents while driving and the economic burden on patients and their caregivers (12). In a follow-up assessment performed in the general population to explore the relationship between EDS and the risk of developing PD, male patients with EDS showed more than three-fold higher risk of PD in comparison with those without EDS (13). Thus, it is important to identify these patients early and provide potential treatment options as soon as possible. Identifying patients with unique clinical features can facilitate analyses of subtype-specific biomarkers and epidemiological and clinical treatments. Although many studies conducted over the past decade have explored the clinical features of EDS in PD patients, there are still some issues that remain unclear and require further research. Moreover, few large-sample studies have focused on studying the clinical features and risk factors of EDS in Chinese patients. Thus, we performed this study to investigate the prevalence, clinical features, and correlates of EDS among PD patients in a Chinese population.

## Methods

### Patients

A total of 1,221 consecutive PD patients in an “off” state were recruited from the clinic or inpatient department of the Department of Neurology, Xiangya Hospital of Central South University (Changsha, China) between February 1, 2017 and April 30, 2018. All patients were diagnosed by a neurological specialist by using the MDS Clinical Diagnostic Criteria (14). Patients with acute psychosis, atypical parkinsonism, and dementia [Mini-Mental State Examination (MMSE) score lower than 24] were excluded. Patients who were unable to finish the questionnaire were also excluded. Written informed consent were obtained from all patients. The study was approved by the Ethics Committee of Xiangya Hospital of Central South University.

### Assessments

All participants underwent clinical evaluation in an “off” state. Clinical data were collected, including demographic data and information regarding lifestyle factors, pesticide exposure, medical history, motor symptoms, and NMS. Levodopa and dopamine agonists were calculated as levodopa equivalent dose (LED) (15). The ratio of the mean tremor score (8 items, including UPDRS II-16, UPDRS III-20, and UPDRS III-21) to the mean postural instability/gait disorder (PIGD) score (5 items, including UPDRS II-13, UPDRS II-14, UPDRS II-15, UPDRS III-29, UPDRS III-30) was used to classify motor subtype. Patients with a ratio value < 1.0 were defined as PIGD, and those with values from 1.0 to 1.5 were categorized as intermediate, while those with values ≥1.5 were classified as tremor dominant (TD) (16). For evaluation of EDS, participants were assessed by Epworth sleepiness scale (ESS), which measures the likelihood of patients falling asleep in eight different situations. All items were rated on a 4-point scale (0–3), with a maximum total score of 24. A higher score demonstrates more severe EDS. Participants were classified as showing EDS if they scored ≥10 on the ESS (17). The severity of disease was measured by the Hoehn-Yahr scale and the unified Parkinson's disease rating scale (UPDRS). Cognitive impairment was evaluated using the MMSE (18). Non-motor symptoms scale (NMSS) items 1–2 and 22–24 were used to assess cardiovascular and urinary symptoms, respectively. A higher score indicates severe symptoms (19). Rapid eye movement sleep behavior disorder (RBD) was evaluated using the REM sleep behavior disorder questionnaire–Hong Kong (RBDQ-HK), which is a valuable tool for screening RBD. The optimal cutoff value for factor 2 of RBDQ-HK was 7/8 with 90% sensitivity and 82% specificity, and that for the RBDQ-HK overall scale was 17 with 85% sensitivity and 81% specificity; subjects were classified as showing RBD when they reached the above score (20). PD-related sleep and nocturnal disability were assessed using the Parkinson's disease sleep scale (PDSS), which includes 15 items that address eight aspects of sleep disturbances in PD, including overall quality of night's sleep (item 1), sleep onset and maintenance insomnia (items 2 and 3), nocturnal restlessness (items 4 and 5), nocturnal psychosis (items 6 and 7), nocturia (items 8 and 9), nocturnal motor symptoms (items 10–13), sleep refreshment (item 14), and daytime dozing (item 15); lower PDSS scores indicate poorer sleep quality (21). Olfactory dysfunction was measured using a hyposmia rating scale (HRS), which is a simple, convenient and reliable method, reaching 70% (60–81%) sensitivity and 85% (65–100%) specificity with a cutoff value of 22.5 (22). The Hamilton rating scale for depression (HAMD-17) was applied to assess depression (23). QoL was evaluated by the 39-item Parkinson's disease questionnaire (PDQ-39) (24).

### Statistical Analysis

For descriptive analyses, conventional statistical parameters including means and standard deviations were used to describe continuous variables, while percentages and frequencies were used for describing categorical variables. The *t*-test or chi-squared test were used to compare the demographic and clinical characteristics between PD patients with and without EDS. Logistic regression analysis was used to evaluate the factors associated with EDS in PD. To ensure the accuracy of the model, variables that were clinically significant or showed a relationship with EDS were included in univariate and multivariate logistic models. In the univariate logistic regression model, after adjusting for age and sex, variables with *P* < 0.2 were considered in the multivariate logistic model. A likelihood ratio backward selection approach was used in the multivariate logistic model. Relationships between PDSS subgroup and ESS score were assayed with partial correlation analysis, and sex, age, and disease duration were adjusted. Statistical analysis was performed using SPSS version 20.0. Significance was considered if *p* < 0.05.

## Results

### Demographic and Medication Data of All PD Patients and Patients With and Without EDS

A total of 1,221 PD patients were included in this study. The demographic data and medication data of all PD patients and PD patients with and without EDS are shown in Table 1. The percentage of male and female patients in our study was 54.1% (661/1,221) and 45.9% (560/1,221), respectively. The mean age of the patients was 61.5 ± 9.9 years. The average age at PD onset and disease duration were 56.4 ± 10.6 years and 5.1 ± 4.5 years, respectively. In terms of educational level, the number of patients with none/primary level, secondary level/high school, and university education were 457 (37.4%), 635 (52.0%), and 129 (10.6%), respectively. The number of patients with a history of smoking, alcohol consumption, tea consumption, and pesticide exposure were 280 (22.9%), 260 (21.3%), 142 (11.6%), and 225 (18.4%), respectively. The average BMI was 22.6 ± 3.7 kg/m^2^. The mean LED of the patients in our study was 325.1 ± 302.8. Patients with EDS were older than those without EDS. The EDS group also showed a higher proportion of alcohol consumption, higher age at PD onset, longer disease duration, and lower educational levels. However, there were no significant differences in smoking, tea consumption, pesticide exposure, BMI, and LED between patients with EDS and those without EDS.

**Table 1:** Demographic and medication data of all PD patients and patients with and without EDS.

**Variable**	**Total**	**With EDS**	**Without EDS**	***P*-value**
	***n* = 1,221**	***n* = 416**	***n* = 805**	
Male sex, *n* (%)^a^	661 (54.1%)	249 (59.9%)	412 (51.2%)	0.004^*^
Age (years)^b^	61.5 ± 9.9	63.8 ± 9.5	60.31 ± 9.9	< 0.001^*^
Age at PD onset, (years)^b^	56.4 ± 10.6	58.1 ± 10.2	55.5 ± 10.7	< 0.001^*^
Disease duration (years)^b^	5.1 ± 4.5	5.7 ± 4.8	4.8 ± 4.2	< 0.001^*^
Educational level, *n* (%)^a^				< 0.001^*^
None/first level, *n* (%)	457 (37.4%)	183 (44.0%)	274 (34.0%)	
Secondary level/high school, *n* (%)	635 (52.0%)	181 (43.5%)	454 (56.4%)	
University, *n* (%)	129 (10.6%)	52 (12.5%)	77 (9.6%)	
Smoking, *n* (%)^a^	280 (22.9%)	100 (24.0%)	180 (22.4%)	0.509
Alcohol, *n* (%)^a^	260 (21.3%)	104 (25.0%)	156 (19.4%)	0.023^*^
Tea drinking, *n* (%)^a^	142 (11.6%)	51 (12.3%)	91 (11.3%)	0.622
Pesticide exposure, *n* (%)^a^	225 (18.4%)	79 (19.0%)	146 (18.1%)	0.715
BMI^b^	22.6 ± 3.7	22.2 ± 4.0	22.6 ± 4.1	0.125
LED^b^	325.1 ± 302.8	338.6 ± 292.0	318.1 ± 308.2	0.265

a *Chi-squared test*.

b *t-test*.

* *Statistically significant (p < 0.05). LED, Levodopa Equivalent Dose; BMI, Body Mass Index*.

### Motor and Non-motor Symptoms in All PD Patients and Patients With and Without EDS

Table 2 summarizes the motor and non-motor symptoms data of all PD patients and the clinical characteristics of PD with EDS and PD without EDS. EDS was noted is 34.1% of the PD patients in our study population. In terms of motor symptoms, patients with EDS had higher Hoehn and Yahr scale scores. In comparison with patients without EDS, the EDS group showed significantly higher scores in UPDRS part I, UPDRS part II, and UPDRS part III. However, there were no significant differences in the initial presentation of motor symptoms, motor subtype, and percentage of wearing off.

**Table 2:** Motor and non-motor symptoms of all PD patients and patients with and without EDS.

**Variable**	**Total**	**With EDS**	**Without EDS**	***P*-value**
	***n* = 1,221**	***n* = 416**	***n* = 805**	
Initial presentation of motor symptoms, *n* (%)^a^	–	–	–	0.199
Tremor	535 (43.8%)	167 (40.1%)	368 (45.7%)	–
Bradykinesia	438 (35.9%)	160 (38.5%)	278 (34.5%)	–
Tremor and bradykinesia	238 (19.5%)	87 (20.9%)	151 (18.8%)	–
Other	10 (0.8%)	2 (0.5%)	8 (1.0%)	–
Hoehn and Yahr stage, *n* (%)^a^	–	–	–	< 0.001^*^
1	178 (14.6%)	45 (10.8%)	133 (16.5%)	–
1.5	161 (13.2%)	41 (9.9%)	120 (14.9%)	–
2	268 (21.9%)	78 (18.8%)	190 (23.6%)	–
2.5	231 (18.9%)	88 (21.2%)	143 (17.8%)	–
3	314 (25.7%)	124 (29.8%)	190 (23.6%)	–
4	53 (4.3%)	32 (7.7%)	21 (2.6%)	–
5	16 (1.3%)	8 (1.9%)	8 (1.0%%)	–
UPDRS part I^b^	2.6 ± 2.0	3.0 ± 2.1	2.3 ± 1.9	< 0.001^*^
UPDRS part II^b^	12.4 ± 6.3	14.4 ± 6.7	11.4 ± 5.9	< 0.001^*^
UPDRS part III^b^	29.3 ± 14.7	32.8 ± 15.4	27.5 ± 14.0	< 0.001^*^
Motor subtype, *n* (%)^a^	–	–	–	0.740
PIGD	784 (64.2%)	271 (65.1%)	513 (63.7%)	–
TD	256 (21.0%)	82 (19.7%)	174 (21.6%)	–
Intermediate	181 (14.8%)	63 (15.1%%)	118 (14.7%)	–
Wearing off, *n* (%)^a^	225 (18.4%)	84 (20.2%)	141 (17.5%)	0.253
MMSE score ^b^	26.7 ± 2.9	26.1 ± 3.1	27.0 ± 2.8	< 0.001^*^
Depression, *n* (%)^a^	437 (35.8%)	192 (46.2%)	245 (30.4%)	< 0.001^*^
ESS score^b^	7.6 ± 6.1	14.8 ± 3.6	3.9 ± 3.1	< 0.001^*^
PDSS score^b^	116.9 ± 24.8	108.3 ± 24.0	121.4 ± 24.1	< 0.001^*^
RBD, *n* (%)^a^	450 (36.9%)	209 (50.2%)	241 (29.9%)	< 0.001^*^
Cardiovascular symptom score^b^	0.87 ± 1.8	1.1 ± 2.1	0.8 ± 1.7	0.001^*^
Urinary symptom score^b^	5.60 ± 6.1	7.0 ± 6.9	4.9 ± 5.5	< 0.001^*^
Constipation, *n* (%)^a^	586 (48.0%)	222 (53.4%)	364 (45.2%)	0.007^*^
Hyposmia, *n* (%)^b^	511 (41.9%)	204 (49.0%)	307 (38.1%)	< 0.001^*^
PDQ-39 score^a^	30.1 ± 24.7	38.7 ± 25.9	25.6 ± 22.8	< 0.001^*^

a *Chi-squared test*.

b *t-test*.

* *Statistically significant (p < 0.05). UPDRS, Unified Parkinson's Disease Rating Scale; PIGD, Postural Instability/Gait Disorder; TD, Tremor Dominant; MMSE, Mini-Mental State Examination; ESS, Epworth Sleepiness Scale; PDSS, Parkinson's Disease Sleep Scale; RBD, Rapid Eye Movement Sleep Behavior Disorder; PDQ-39, 39-item Parkinson's Disease Questionnaire*.

When comparing NMS between patients with and without EDS, we found that participants with EDS had higher scores for ESS, cardiovascular symptoms, urinary symptoms, and PDQ-39, but lower scores for MMSE and PDSS. Furthermore, in comparison with patients without EDS, those with EDS showed a significantly higher proportion of depression, RBD, constipation, and hyposmia.

### Logistic Regression Analysis of Factors Associated With EDS

Table 3 shows the factors associated with EDS. The logistic regression analysis indicated that male sex, age, cognitive impairment, PD-related sleep problems, RBD, and worse QoL were associated with EDS in PD patients.

**Table 3:** Logistic regression analysis of factors associated with EDS.

**Variable**	**Univariate *P*-value**	**Multivariate OR (95% CI)**	**Multivariate *P*-value**
Sex	0.004	0.605(0.466–0.785)	< 0.001*^*^
Age	< 0.001	1.021(1.007–1.035)	0.004^*^
Educational level	0.127	—	NS
Age at PD onset	0.001	—	NS
Disease duration	0.001	—	NS
LED	0.290	—	Not included
Initial presentation of motor symptoms	0.570	—	Not included
Hoehn and Yahr stage	< 0.001	—	NS
UPDRS part I	< 0.001	—	NS
UPDRS part II	< 0.001	—	NS
UPDRS part III	< 0.001	—	NS
Motor subtype	0.879	—	Not included
MMSE score	< 0.001	0.950(0.907–0.995)	0.030^*^
Depression	< 0.001	—	NS
PDSS score	< 0.001	0.988(0.982–0.993)	< 0.001^*^
RBD	< 0.001	1.627(1.247–2.121)	< 0.001^*^
Cardiovascular symptom score	0.009	—	NS
Urinary symptom score	< 0.001	—	NS
Constipation	0.306	—	Not included
Hyposmia	0.005	—	NS
PDQ-39 score	< 0.001	1.012(1.006–1.018)	< 0.001^*^

*All univariate analyses were adjusted for age and sex. The multivariate analysis forced age and sex into the model. NS, not significant; Not included, variables with P > 0.2 in the univariate logistic regression model. ^−^Variables not included in the multivariate logistic model or variables with P > 0.05 in the multivariate logistic model*.

* *Statistically significant (p < 0.05). LED, Levodopa Equivalent Dose; UPDRS, Unified Parkinson's Disease Rating Scale; MMSE, Mini-Mental State Examination; PDSS, Parkinson's Disease Sleep Scale; RBD, Rapid Eye Movement Sleep Behavior Disorder; PDQ-39, 39-item Parkinson's Disease Questionnaire*.

### The Relationship Between PD-Related Nocturnal Sleep Problems and EDS in PD Patients

**Figure 1:**
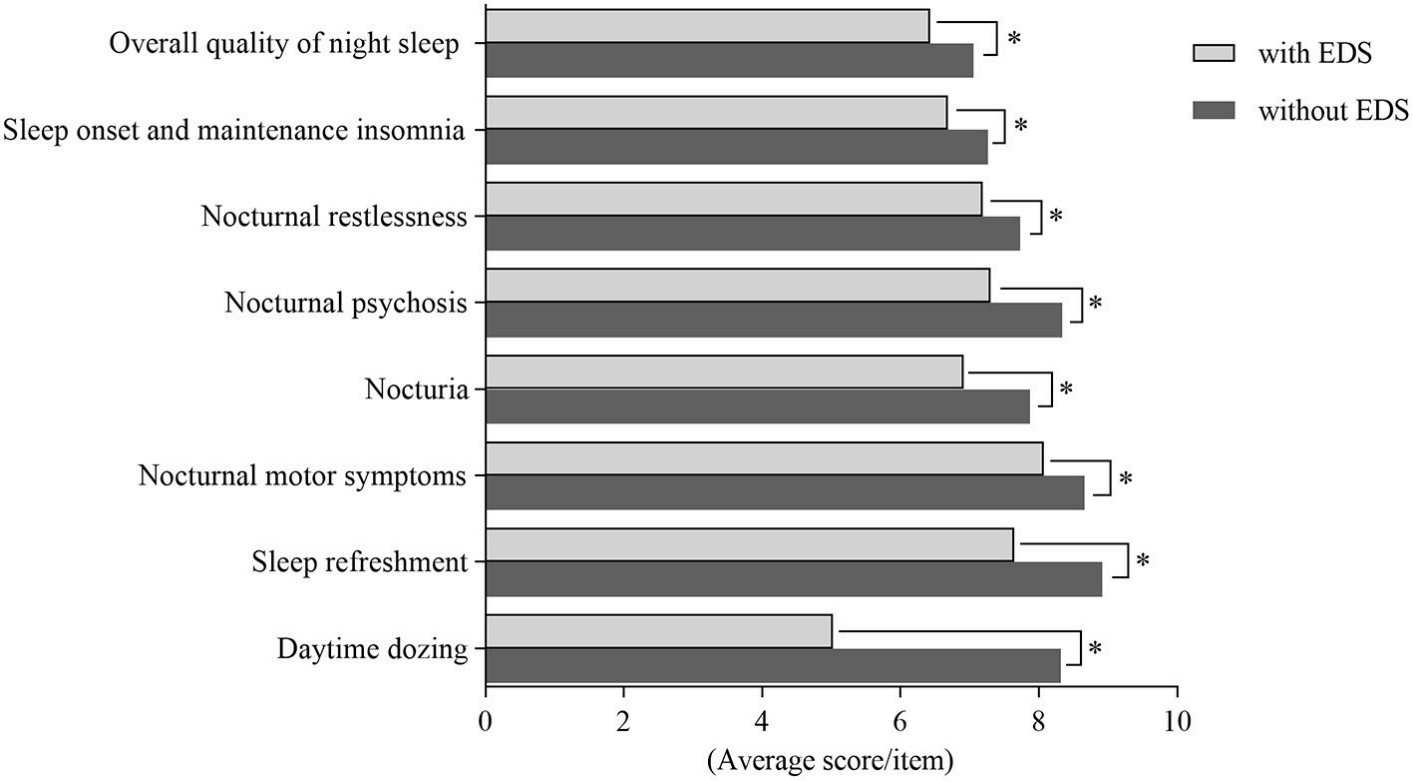
Comparing subgroups of Parkinson's Disease Sleep Scale (PDSS) among patients with EDS and without EDS. There were significant differences in aspects of overall quality of night sleep, sleep onset and maintenance insomnia, nocturnal restlessness, nocturnal psychosis, nocturia, nocturnal motor symptoms, sleep refreshment and daytime dozing between patients with EDS and patients without EDS. ^*^Statistically significant (*p* < 0.05).

The findings showed that patients with EDS had poorer quality of sleep than those without EDS. In order to thoroughly study the relationship between EDS and PD-related nocturnal sleep problems, we compared the PDSS subgroup scores between patients with and without EDS. We also assessed correlations between the ESS scores and PDSS subgroup scores. There were significant differences in the overall quality of night sleep, sleep onset and maintenance insomnia, nocturnal restlessness, nocturnal psychosis, nocturia, nocturnal motor symptoms, sleep refreshment, and daytime dozing between two groups. The differences in PDSS subgroup scores between patients with and without EDS are shown in Figure 1.

Further, analysis of the ESS scores and PDSS subgroup scores showed weak to moderate correlations in the following aspects: overall quality of night sleep (*r* = −0.118, *p* < 0.001), sleep onset and maintenance insomnia (*r* = −0.073, *p* = 0.011), nocturnal restlessness (*r* = −0.080, *p* = 0.005), nocturnal psychosis (*r* = −0.175, *p* < 0.001), nocturia (*r* = −0.122, *p* < 0.001), nocturnal motor symptoms (*r* = −0.138, *p* < 0.001), sleep refreshment (*r* = −0.171, *p* < 0.001), and daytime dozing (*r* = −0.536, *p* < 0.001). The results of these, analysis are shown in Table 4.

**Table 4:** Correlation coefficient between ESS scores and PDSS subgroup scores.

**Subgroups**	**Correlation coefficient**	***P*-value**
Overall quality of night sleep	−0.118	< 0.001^*^
Sleep onset and maintenance insomnia	−0.073	0.011^*^
Nocturnal restlessness	−0.080	0.005^*^
Nocturnal psychosis	−0.175	< 0.001^*^
Nocturia	−0.122	< 0.001^*^
Nocturnal motor symptoms	−0.138	< 0.001^*^
Sleep refreshment	−0.171	< 0.001^*^
Daytime dozing	−0.536	< 0.001^*^

* *Statistically significant (p < 0.05)*.

## Discussion

To the best of our knowledge, this study is the largest reported cross-sectional investigation of the incidence, clinical characteristics, and associated factors of EDS among PD patients in a Chinese population. A wide range of evaluation methods were used to assess various factors that can potentially influence EDS. The prevalence of EDS was 34.1% in our study. Our findings showed significant differences in demographic factors and motor and non-motor symptoms between patients with and without EDS. Moreover, we revealed that EDS was associated with many variables.

The prevalence of EDS is lower than that reported in other population studies (25, 26). This difference may be attributed to the differences in methodology and patient cohort demographics and cultural factors that may have influenced questionnaire interpretation. Patients with EDS showed specific clinical parameters. In comparison with patients without EDS, those with EDS included a higher proportion of male patients, consistent with a previous study (8). Moreover, male gender was associated with EDS in our study, whereas in a general population, the contribution of male gender in EDS was not confirmed (27). These discrepancies possibly indicate that there were differences in susceptibility for males with PD. Interestingly, we found that patients with EDS were older, and this was a contributor to EDS. The aging process led to macro-level changes in the sleep structure and micro-level changes in the sleep architecture, such as advanced sleep-phase disturbances and reductions in slow-wave activity (28). This discovery suggested that age processing combined with disease-related biologic changes in certain areas of the brain induce or promote EDS. Thus, long-term follow-up studies to assess the contribution of age to EDS are essential. Notably, patients with EDS showed a higher age at PD onset than those without EDS. It is possible that patients with different etiologies for developing EDS show different ages of onset (7). Environmental factors were not associated with EDS in PD. This result illustrated that environmental factors are possibly only related to the occurrence of PD other than EDS.

The effect of antiparkinsonian medications on EDS is controversial and complex. Levodopa and dopamine agonists significantly improve the patient's motor symptoms, but they may also increase the incidence of other clinical manifestations, such as EDS and dyskinesia (29, 30). Dopamine has biphasic effects on wakefulness (31). Furthermore, many studies have shown that classic and newer stimulant medications exert their effect through dopaminergic mechanisms, which may be independent of their effects on motor activity (32). A previous study revealed that LED was correlated with EDS. The ESS score significantly increased and the mean multiple sleep latency test (MSLT) score decreased after treatments with dopaminergic drugs (33). However, our study has shown that there was no significant difference in LED between patients with EDS and those without EDS, but a tendency toward a higher LED in the PD with EDS group was found. This result was consistent with another study that reported no significant associations between dopaminergic drugs and either subjective or objective EDS (34). Bliwise et al. indicated that increasing dosages of dopamine agonists were positively associated with less daytime alertness, whereas higher levels of levodopa were related to higher levels of alertness (35). This is likely because patients were predominantly treated with levodopa and the doses of agonists were relatively low in our population, so there was no significant difference in LED. The effects of dopaminergic drugs on EDS need further study.

Expectedly, the EDS group showed longer disease duration, more advanced disease, and higher UPDRS score than the PD without EDS group in our population, indicating that EDS may be associated with more severe neurodegeneration within the ascending arousal system of the brainstem. Prior longitudinal studies indicated that the prevalence of EDS increases as the disease progresses. In a study consisting of 232 patients, the prevalence of EDS increased from 5.6% at the baseline to 22.5% at the 4-year follow-up and 40.8% at the 8-year follow-up (29). Braak et al. reported that Lewy bodies emerged from the dorsal motor vagal nucleus and progressed in an ascending course with the progression of disease (36). The pedunculopontine nucleus (PPN), locus coeruleus (LC), nucleus magnocellularis, and tegmental area are significant parts of the ascending arousal systems in brainstem. When these areas involved in regulating sleep and awareness are affected, sleep disorders including EDS may occur (37, 38).

The clinical NMS showing differences between patients with and without EDS in our population included cognitive impairment, depression, PD-related sleep disorder, RBD, cardiovascular symptoms, urinary symptoms, constipation, and hyposmia. Cognitive impairment is a common feature of PD and a well-known contributor of EDS (39). Jennifer et al reported that the ESS scores of patients with normal cognition (PD-NC), mild cognitive impairment (PD-MCI), and dementia (PDD) were significantly different. ESS scores was associated with cognitive impairment, including the cognitive domains of attention/working memory, executive function, memory, and visuospatial function (25). Our results indicated that the EDS group had lower MMSE scores and this was associated with EDS, which was consistent with the data from other PD populations. EDS in PD implies neuronal loss and Lewy body aggregation in the brainstem, hypothalamus, basal forebrain regions, and thalamus along with alterations in the cholinergic, monoaminergic, dopaminergic, and histaminergic systems. It is noteworthy that similar neuroanatomical regions and neurotransmitter systems that regulate sleep-wake cycle may be connected with attention, executive function, learning, and memory (40, 41). Depression frequently occurs in PD patients, with the prevalence of depression ranging from 2.7 to 90% (42). Our study indicated that patients with EDS had more severe depression than those without EDS and that this was associated with EDS. This study is supported by prior studies that have reported a strong association between depression and EDS (43), but the specific pathophysiological mechanism underlying this association remains unknown. Therefore, in PD patients with EDS, it is necessary to focus on mood disorders such as depression and use neuropsychological scales to assess the patients' mood. Sleep architecture is overtly disturbed in PD patients due to disease-related pathological changes. Polysomnography showed shorter total sleep time, lower sleep efficiency, and reduced slow-wave and REM sleep time in PD patients (44, 45). Nocturnal problems have a significant impact on PD patients. Previous studies indicated that EDS may be correlated with poor nocturnal sleep, such as restless legs syndrome, nocturnal motor symptoms, and nocturia (46–48). Notably, in our population, we found that the PDSS total and subgroup scores in patients with EDS were lower than those in patients without EDS, and a low PDSS score was related to EDS. Furthermore, correlation analyses confirmed this relationship between EDS and PD-related nocturnal sleep disturbance. However, this correlation was weak, indicating that EDS may be a consequence of poor night sleep. RBD is a common prodromal maker of PD. In a prior series, EDS was significantly correlated with iRBD (idiopathic RBD) in patients aged < 50 years (49), and a study consisting of 432 PD participants and 196 healthy controls revealed that RBD was closely associated with EDS in univariate analysis, but the association became insignificant in multivariate analysis (50). In contrast, in our population, RBD was significant both in univariate and multivariate analyses. This is likely because patients in our population were in advanced disease stage than those in prior studies, which included participants in drug-naïve and early stages.

Furthermore, the relationship between EDS and cardiovascular symptoms, urinary symptoms, and hyposmia has not been systematically studied in large samples. Our result demonstrated that the EDS group had more severe autonomic and olfactory dysfunction, all of which may associated with the greater spread of neurodegeneration within the areas controlling autonomic function and olfaction (51). Of note, the autonomic nervous system is controlled by a wide range of central and peripheral neural networks, both of which are involved in PD and contribute to nocturia and are subsequently accompanied by nocturnal sleep disorders or daytime sleepiness (52, 53). Indeed, EDS has a negative influence on the QoL of patients and increases the burden on caregivers. Our data corroborate the findings of previous studies that suggested that QoL impairment was more common in PD patients with EDS than in those without EDS, and that poorer QoL was associated with EDS, as evaluated using the PDQ-39 scale (54, 55).

The strengths of this study are the large sample size and the use of detailed medical record information. The limitation of our study is a lack of subjective measures to assess sleep disorders. Besides, the nature of a cross-sectional study was another limitation. Further prospective studies are necessary to elucidate the clinical features and risk factors of EDS.

In conclusion, we conducted the largest sample study to systematically investigate the prevalence, clinical features, and correlates of EDS in a Chinese population of PD patients. Our finding indicated that PD with EDS presented with more severe motor symptoms and NMS than PD without EDS. Further, our findings demonstrated that male sex, age, cognitive impairment, PD-related sleep problems, RBD, and worse QoL were associated with EDS in PD patients.

## Author Contributions

JG and BT conceived the study. YX, QX, QS, YT, and LF carried out the analysis and interpretation of the data. ZW, YY, JT edited the manuscript. XY, BT, JG discussed and revised the manuscript. All authors gave final approval for the manuscript to be published.

### Conflict of Interest Statement

The authors declare that the research was conducted in the absence of any commercial or financial relationships that could be construed as a potential conflict of interest.

## References

[1] Yang Y Tang BS Weng L Li N Shen L Wang J. Genetic identification is critical for the diagnosis of parkinsonism: a chinese pedigree with early onset of parkinsonism. PLoS ONE (2015) 10:e0136245. 10.1371/journal.pone.0136245 26295349 PMC4546630

[2] Guo JF Zhang L Li K Mei JP Xue J Chen J. Coding mutations in NUS1 contribute to Parkinson's disease. Proc Natl Acad Sci USA. (2018) 115:11567–72. 10.1073/pnas.1809969115 30348779 PMC6233099

[3] Yang Y Tang BS Guo JF. Parkinson's disease and cognitive impairment. Parkinsons Dis. (2016) 2016:6734678. 10.1155/2016/6734678 28058128 PMC5183770

[4] Hughes KC Gao X Baker JM Stephen C Kim IY Valeri L. Non-motor features of Parkinson's disease in a nested case-control study of US men. J Neurol Neurosurg Psychiatry (2018) 89:1288–95. 10.1136/jnnp-2018-318275 30076266

[5] Suzuki K Okuma Y Uchiyama T Miyamoto M Sakakibara R Shimo Y. Impact of sleep-related symptoms on clinical motor subtypes and disability in Parkinson's disease: a multicentre cross-sectional study. J Neurol Neurosurg Psychiatry (2017) 88:953–9. 10.1136/jnnp-2017-316136 28847794 PMC5740547

[6] Lin YY Chen RS Lu CS Huang YZ Weng YH Yeh TH. Sleep disturbances in Taiwanese patients with Parkinson's disease. Brain Behav. (2017) 7:e00806. 10.1002/brb3.806 29075566 PMC5651390

[7] Zhu K Van Hilten JJ Marinus J. Course and risk factors for excessive daytime sleepiness in Parkinson's disease. Parkinsonism Relat Disord. (2016) 24:34–40. 10.1016/j.parkreldis.2016.01.020 26846609

[8] Tholfsen LK Larsen JP Schulz J Tysnes OB Gjerstad MD. Development of excessive daytime sleepiness in early Parkinson disease. Neurology (2015) 85:162–8. 10.1212/WNL.0000000000001737 26085603

[9] Breen DP Williams-Gray CH Mason SL Foltynie T Barker RA. Excessive daytime sleepiness and its risk factors in incident Parkinson's disease. J Neurol Neurosurg Psychiatry (2013) 84:233–4. 10.1136/jnnp-2012-304097 23184153

[10] Verbaan D Van Rooden SM Visser M Marinus J Van Hilten JJ. Nighttime sleep problems and daytime sleepiness in Parkinson's disease. Mov Disord. (2008) 23:35–41. 10.1002/mds.21727 17960797

[11] Amara AW Chahine LM Caspell-Garcia C Long JD Coffey C Hogl B. Longitudinal assessment of excessive daytime sleepiness in early Parkinson's disease. J Neurol Neurosurg Psychiatry (2017) 88:653–62. 10.1136/jnnp-2016-315023 28554959 PMC7282477

[12] Ueno T Kon T Haga R Nishijima H Tomiyama M. Motor vehicle accidents in Parkinson's disease: a questionnaire study. Acta Neurol Scand. (2018) 137:218–23. 10.1111/ane.12849 28948617

[13] Abbott RD Ross GW White LR Tanner CM Masaki KH Nelson JS. Excessive daytime sleepiness and subsequent development of Parkinson disease. Neurology (2005) 65:1442–6. 10.1212/01.wnl.0000183056.89590.0d 16275833

[14] Postuma RB Berg D Stern M Poewe W Olanow CW Oertel W. MDS clinical diagnostic criteria for Parkinson's disease. Mov Disord. (2015) 30:1591–601. 10.1002/mds.26424 26474316

[15] Tomlinson CL Stowe R Patel S Rick C Gray R Clarke CE. Systematic review of levodopa dose equivalency reporting in Parkinson's disease. Mov Disord. (2010) 25:2649–53. 10.1002/mds.23429 21069833

[16] Stebbins GT Goetz CG Burn DJ Jankovic J Khoo TK Tilley BC. How to identify tremor dominant and postural instability/gait difficulty groups with the movement disorder society unified Parkinson's disease rating scale: comparison with the unified Parkinson's disease rating scale. Mov Disord. (2013) 28:668–70. 10.1002/mds.25383 23408503

[17] Johns MW. A new method for measuring daytime sleepiness: the Epworth sleepiness scale. Sleep (1991) 14:540–5. 10.1093/sleep/14.6.540 1798888

[18] Folstein MF Folstein SE Mchugh PR. Mini-mental state. A practical method for grading the cognitive state of patients for the clinician. J Psychiatr Res. (1975) 12:189–98. 10.1016/0022-3956(75)90026-6 1202204

[19] Chaudhuri KR Martinez-Martin P Brown RG Sethi K Stocchi F Odin P. The metric properties of a novel non-motor symptoms scale for Parkinson's disease: results from an international pilot study. Mov Disord. (2007) 22:1901–11. 10.1002/mds.21596 17674410

[20] Shen SS Shen Y Xiong KP Chen J Mao CJ Huang JY. Validation study of REM sleep behavior disorder questionnaire-Hong Kong (RBDQ-HK) in east China. Sleep Med. (2014) 15:952–8. 10.1016/j.sleep.2014.03.020 24938584

[21] Chaudhuri KR Pal S Dimarco A Whately-Smith C Bridgman K Mathew R. The Parkinson's disease sleep scale: a new instrument for assessing sleep and nocturnal disability in Parkinson's disease. J Neurol Neurosurg Psychiatry (2002) 73:629–35. 10.1136/jnnp.73.6.629 12438461 PMC1757333

[22] Millar Vernetti P Perez Lloret S Rossi M Cerquetti D Merello M Validation of a new scale to assess olfactory dysfunction in patients with Parkinson's disease. Parkinsonism Relat Disord. (2012) 18:358–61. 10.1016/j.parkreldis.2011.12.001 22227345

[23] Leentjens AF Verhey FR Lousberg R Spitsbergen H Wilmink FW. The validity of the Hamilton and Montgomery-Asberg depression rating scales as screening and diagnostic tools for depression in Parkinson's disease. Int J Geriatr Psychiatry (2000) 15:644–9. 10.1002/1099-1166(200007)15:7<644::AID-GPS167>3.0.CO;2-L 10918346

[24] Peto V Jenkinson C Fitzpatrick R Greenhall R. The development and validation of a short measure of functioning and well being for individuals with Parkinson's disease. Qual Life Res. (1995) 4:241–8. 10.1007/BF02260863 7613534

[25] Goldman JG Ghode RA Ouyang B Bernard B Goetz CG Stebbins GT. Dissociations among daytime sleepiness, nighttime sleep, and cognitive status in Parkinson's disease. Parkinsonism Relat Disord. (2013) 19:806–11. 10.1016/j.parkreldis.2013.05.006 23735187 PMC3729741

[26] Poryazova R Benninger D Waldvogel D Bassetti CL. Excessive daytime sleepiness in Parkinson's disease: characteristics and determinants. Eur Neurol. (2010) 63:129–35. 10.1159/000276402 20090346

[27] Baldwin CM Kapur VK Holberg CJ Rosen C Nieto FJ. Associations between gender and measures of daytime somnolence in the sleep heart health study. Sleep (2004) 27:305–11. 10.1093/sleep/27.2.305 15124727

[28] Mander BA Winer JR Walker MP. Sleep and human aging. Neuron (2017) 94:19–36. 10.1016/j.neuron.2017.02.004 28384471 PMC5810920

[29] Gjerstad MD Alves G Wentzel-Larsen T Aarsland D Larsen JP Excessive daytime sleepiness in Parkinson disease: is it the drugs or the disease? Neurology (2006) 67:853–8. 10.1212/01.wnl.0000233980.25978.9d 16966550

[30] Zhang YH Tang BS Song CY Xu Q Lou MX Liu ZH. The relationship between the phenotype of Parkinson's disease and levodopa-induced dyskinesia. Neurosci Lett. (2013) 556:109–12. 10.1016/j.neulet.2013.10.018 24135335

[31] Hauser RA Gauger L Anderson WM Zesiewicz TA. Pramipexole-induced somnolence and episodes of daytime sleep. Mov Disord. (2000) 15:658–63. 10.1002/1531-8257(200007)15:4<658::AID-MDS1009>3.0.CO;2-N 10928575

[32] Wisor JP Nishino S Sora I Uhl GH Mignot E Edgar DM. Dopaminergic role in stimulant-induced wakefulness. J Neurosci. (2001) 21:1787–94. 10.1523/JNEUROSCI.21-05-01787.2001 11222668 PMC6762940

[33] Kaynak D Kiziltan G Kaynak H Benbir G Uysal O. Sleep and sleepiness in patients with Parkinson's disease before and after dopaminergic treatment. Eur J Neurol. (2005) 12:199–207. 10.1111/j.1468-1331.2004.00971.x 15693809

[34] Cochen De Cock V Bayard S Jaussent I Charif M Grini M Langenier MC Daytime sleepiness in Parkinson's disease: a reappraisal. PLoS ONE (2014) 9:e107278 10.1371/journal.pone.0107278 25198548 PMC4157859

[35] Bliwise DL Trotti LM Wilson AG Greer SA Wood-Siverio C Juncos JJ. Daytime alertness in Parkinson's disease: potentially dose-dependent, divergent effects by drug class. Mov Disord. (2012) 27:1118–24. 10.1002/mds.25082 22753297 PMC3589103

[36] Braak H Del Tredici K Rub U De Vos RA Jansen Steur EN Braak E. Staging of brain pathology related to sporadic Parkinson's disease. Neurobiol Aging (2003) 24:197–211. 10.1016/S0197-4580(02)00065-9 12498954

[37] Diederich NJ Mcintyre DJ. Sleep disorders in Parkinson's disease: many causes, few therapeutic options. J Neurol Sci. (2012) 314:12–9. 10.1016/j.jns.2011.10.025 22118862

[38] Saper CB Chou TC Scammell TE. The sleep switch: hypothalamic control of sleep and wakefulness. Trends Neurosci. (2001) 24:726–31. 10.1016/S0166-2236(00)02002-6 11718878

[39] Wang YQ Tang BS Yan XX Chen ZH Xu Q Liu ZH. A neurophysiological profile in Parkinson's disease with mild cognitive impairment and dementia in China. J Clin Neurosci. (2015) 22:981–5. 10.1016/j.jocn.2014.11.030 25890772

[40] Braak H Ghebremedhin E Rub U Bratzke H Del Tredici K. Stages in the development of Parkinson's disease-related pathology. Cell Tissue Res. (2004) 318:121–34. 10.1007/s00441-004-0956-9 15338272

[41] Emre M Aarsland D Brown R Burn DJ Duyckaerts C Mizuno Y. Clinical diagnostic criteria for dementia associated with Parkinson's disease. Mov Disord. (2007) 22:1689–707; quiz 1837. 10.1002/mds.21507 17542011

[42] Reijnders JS Ehrt U Weber WE Aarsland D Leentjens AF. A systematic review of prevalence studies of depression in Parkinson's disease. Mov Disord. (2008) 23, 183–9; quiz 313. 10.1002/mds.21803 17987654

[43] Kurtis MM Rodriguez-Blazquez C Martinez-Martin P. Relationship between sleep disorders and other non-motor symptoms in Parkinson's disease. Parkinsonism Relat Disord. (2013) 19:1152–5. 10.1016/j.parkreldis.2013.07.026 23953775

[44] Shpirer I Miniovitz A Klein C Goldstein R Prokhorov T Theitler J. Excessive daytime sleepiness in patients with Parkinson's disease: a polysomnography study. Mov Disord. (2006) 21:1432–8. 10.1002/mds.21002 16773617

[45] Selvaraj VK Keshavamurthy B. Sleep dysfunction in Parkinson's disease. J Clin Diagn Res. (2016) 10:Oc09–12. 10.7860/JCDR/2016/16446.7208 27042494 PMC4800560

[46] Bhat S Chokroverty S. Hypersomnia in neurodegenerative diseases. Sleep Med Clin. (2017) 12:443–60. 10.1016/j.jsmc.2017.03.017 28778241

[47] Lees AJ Blackburn NA Campbell VL. The nighttime problems of Parkinson's disease. Clin Neuropharmacol. (1988) 11:512–9. 10.1097/00002826-198812000-00004 3233589

[48] Ferreira JJ Desboeuf K Galitzky M Thalamas C Brefel-Courbon C Fabre N. Sleep disruption, daytime somnolence and 'sleep attacks' in Parkinson's disease: a clinical survey in PD patients and age-matched healthy volunteers. Eur J Neurol. (2006) 13:209–14. 10.1111/j.1468-1331.2006.01262.x 16618334

[49] Bonakis A Howard RS Ebrahim IO Merritt S Williams A. REM sleep behaviour disorder (RBD) and its associations in young patients. Sleep Med. (2009) 10:641–5. 10.1016/j.sleep.2008.07.008 19109063

[50] Simuni T Caspell-Garcia C Coffey C Chahine LM Lasch S Oertel WH. Correlates of excessive daytime sleepiness in *de novo* Parkinson's disease: a case control study. Mov Disord. (2015) 30:1371–81. 10.1002/mds.26248 26095202 PMC4822999

[51] Sulzer D Surmeier DJ Neuronal vulnerability, pathogenesis, and Parkinson's disease. Mov Disord. (2013) 28:715–24. 10.1002/mds.25187 23589357

[52] Weerkamp NJ Tissingh G Poels PJ Zuidema SU Munneke M Koopmans RT. Nonmotor symptoms in nursing home residents with Parkinson's disease: prevalence and effect on quality of life. J Am Geriatr Soc. (2013) 61:1714–21. 10.1111/jgs.12458 24117286

[53] Kingsbury AE Bandopadhyay R Silveira-Moriyama L Ayling H Kallis C Sterlacci W. Brain stem pathology in Parkinson's disease: an evaluation of the Braak staging model. Mov Disord. (2010) 25:2508–15. 10.1002/mds.23305 20818670

[54] Gjerstad MD Aarsland D Larsen JP. Development of daytime somnolence over time in Parkinson's disease. Neurology (2002) 58:1544–6. 10.1212/WNL.58.10.1544 12034797

[55] Ghorayeb I Loundou A Auquier P Dauvilliers Y Bioulac B Tison F. A nationwide survey of excessive daytime sleepiness in Parkinson's disease in France. Mov Disord. (2007) 22:1567–72. 10.1002/mds.21541 17534963

